# ‘Who are you today?’ Problems of identity in psychiatry

**DOI:** 10.1192/bji.2020.6

**Published:** 2020-08

**Authors:** Charles Foster

**Affiliations:** Visiting Professor, Faculty of Law, University of Oxford, UK. Email: charles.foster@law.ox.ac.uk

**Keywords:** Dementia, dissociative disorders, ethics, psychiatry and law

## Abstract

Our attributes change. Sometimes they are changed so dramatically (for instance by organic brain disease, traumatic brain injury or psychiatric disease) that it is hard to see any significant continuity with the premorbid person. Sometimes this can have important ethical and legal consequences, but the problems are often ignored. This article highlights some of the difficulties.

## Can we define a ‘right mind’?

‘What's your job?’, I asked a very experienced psychiatrist. ‘Simple’, she said. ‘It's to put the patient back into his right mind’.

Whatever one says about that statement, it is not simple. It makes some tectonic assumptions. It assumes that the human mind is like a tibia and, like a tibia, can be broken and then reset. It assumes that there is such a state as a ‘right mind’ – or at least a mind that is less un-right than others. It may (and probably does) make a normative assumption about what constitutes a right mind: an optimally ‘right mind’ is probably the sort of mind that the psychiatrist herself would wish to have: the sort of mind that enables polite participation in Western society. The use of the word ‘back’ implies that the ‘right mind’ is the one that previously existed – although if the previous mind was less like the psychiatrist's own mind than the one the patient now has, this notion will no doubt be quietly dropped.

It implies that minds are immutable except by disease. Indeed, it tends to the conclusion that ‘mind’ is really another word for ‘soul’ and that psychiatrists are really ‘soul doctors’, exorcising the demons of psychiatric illness and making the soul clean and habitable again. It assumes (often reasonably, since the patient will generally have attended voluntarily) that the patient consents to being put back into their ‘right mind’, and accordingly that the un-right mind is judged by the patient – by whatever criteria – to be an uncomfortable or painful place.

The normative elements of all this are consonant with the usual entries in the clinical notes: ‘Dressed appropriately’ (according to whose canon of fashion?). ‘Normal content and delivery of speech’ (Shakespeare would fail on the content, King Edward VII on the delivery). And so on.None of this is to deny that there is genuine psychopathology. I am not romanticising mental illness (although some psychoses need urgent rehabilitation). And of course some psychopathology will manifest in strange dress, unusual speech patterns and a shift from the patient's own behavioural baseline. Yet the assumptions about the existence and immutability of the normal mind and its identification with the self or the soul need to be examined strenuously. They are rarely examined at all, whether by doctors, lawyers, courts, politicians or theologians.

## Assumptions about the mutability of the self

The assumptions about the normativity of the mind/self/soul (I will refer to them all as the ‘self’) are politically troubling. I leave them for another day. But the assumptions about the mutability of the self are clinically, ethically and legally worrying, not least because disease (the diagnosis of which hands some terrifying powers to clinicians and other decision makers) is *defined* as that which interferes with the normal immutability of the self. If the self is not truly immutable in its un-diseased state, then the definition of disease is defective, and the powers that come with the diagnosis are erroneously exercised.

Yet there are reasons to question our traditional model of the self. Our attributes – including our preferences – change with time, with life experience, with the amount of sleep we have had and with the amount of wine we have drunk. Psychiatry picks rather arbitrarily on a particular suite of attributes, asserting that they are ontologically significant, and also decides, again arbitrarily, how much of an attribute has to be lost or gained in order to justify the label ‘pathology’. The arbitrariness of this process takes us back to the concerns about normativity.

The law is even less nuanced. In most jurisdictions it tends to assume that the person named on the birth certificate is also the person named on the death certificate, and that they are the same person at all points in between.^1^ Where capacity is lost or truncated, the law – depending on jurisdiction – uses one of three devices in order to facilitate decision-making on behalf of the incapacitous patient. It asks what is in the best interests of the patient, or what the patient would have done had he or she found themselves in that position (substituted judgement) or, if a proxy decision maker has been validly appointed, the proxy's word rules (it being assumed that the proxy will make the decision that the patient would have made). The best interests and substituted judgement tests are often conflated – notably and explicitly in the UK.

The significance of this for present purposes is that the starting – and often the finishing – point is the presumed wishes of the patient. Even when her attributes have been dramatically changed by (for example) organic brain injury, the law clings onto the presumption that ‘she’ (now represented by the corpus of her presumed wishes and interests) exists effectively unchanged. Clinicians are directed by the law to treat not the patient in front of them, but a legal fiction: a ghost. It is legally tricky for them to say: ‘We have a new person here. There is little real, clinically significant continuity between the patient in the consulting room and the ex-person whose body she happens to occupy’. They are directed to presume continuity, and that presumed continuity is often decisive for medical decision-making.

## The poverty of the zeitgeist, revealed in clinical conundrums

These observations are often made in the context of a diatribe against the hegemony of autonomy in medical law and ethics. I sympathise with those diatribes, and have often made them myself, but they do not capture all the philosophical and legal poverty of the zeitgeist.

It is in psychiatric and other medical case notes that we see the greatest challenges to the model. Three examples follow. They are all fictitious (though based on real cases). In no case do I offer an answer: all I can do is highlight problems and hope to make clinicians’ jobs even harder than they are already.

### Permanent vegetative state

David has a diagnosis of permanent vegetative state (PVS). If the diagnosis is right, it means that he has not, and will never have, any awareness of anything at all. His clinicians and relatives are agreed that his nasogastric feeding should be stopped, allowing him to die.

But a psychiatrist intervenes, pointing out that all that has been demonstrated is that David has none of the physiological footprints of consciousness. Consciousness, she contends, is a very overrated commodity. Very little of what drives us, and very little of what constitutes what we say is distinctively *us,* is conscious. That, after all, is the canonical premise of psychoanalysis. For all the clinicians know, David might be having the time of his life, released from all the burdens of consciousness, connected with his unconscious and subconscious in the way that expensive therapy is designed to achieve. That connection (so far as we know) demands biological life, and so unless it can be conclusively demonstrated that life is a detriment for David, he should continue to be fed.^2–4^

Does one need to have consciousness, or the possibility of eventually restored consciousness, in order to have a patient? If so, to *whom* is psychoanalytic treatment directed? The name ‘David’ is on the clinical notes, but does ‘David’ really mean ‘David's consciousness’?

‘If David dies’, say his parents and siblings, who spend hours each day at his bedside (and are curiously philosophically educated), ‘part of us will die too. He is part of us, and we are part of him. Humans are porous. They bleed into one another. We're not atomistic billiard balls’.

This suggestion would seem obvious in many non-Western cultures, where the idea of the extended or distributed self is common^5,6^ (though perhaps overstated in the literature).^7–9^ Does it or should it mean that the relatives should be regarded as patients too – because they are part of David and/or because they will be affected?

‘If David lives’, the clinicians reply, ‘many other patients will die. The resources spent keeping him alive are enormous. We have to think of other actual and potential patients in making our decisions. Our duty is to patient*s* plural. That's proper communitarian thinking for you’.

Are the clinicians right?

### Dissociative identity disorder

Dr Jekyll and Mr Hyde cohabit inside a man whose legal name is John. Hyde, when he gets the upper hand, goes out and attacks strangers. Jekyll sometimes discovers what Hyde has done and begs a psychiatrist for treatment which will have the effect of chemically killing Hyde. John, if he is a separate person, agrees that this should be done, because he does not want his body to be securely detained. Assuming such treatment to be possible, should the psychiatrist agree? Or would this amount to unlawful killing? Isn't Hyde a patient too? And shouldn't his interests be considered? Hyde might be depraved, but that doesn't mean that he can be extrajudicially executed.

### Dementia

Salim fears Alzheimer's dementia. He makes it clear in a legally binding document that if he is diagnosed with it, he does not want any life-saving or -sustaining treatment. He gets Alzheimer's dementia. It seems to be the best thing that has happened to him. He is apparently happier than ever before. He contracts a life-threatening chest infection. If he is not given amoxycillin he will die. Should he be treated?

Who is the patient whose interests should be considered? Salim 1 (who feared Alzheimer's dementia) seems to have died – although his name is on the notes and the heart that was his is beating. None of his significant attributes survives. Salim 2 seems to be connected to Salim 1 only by reason of his occupancy of a body composed of some of the same cells, and because the law insists that Salim 1 and Salim 2 are the same.

Sometimes it is hard to identify your patients.
